# Relationship between the magnitude of haemoglobin changes and long-term mortality in patients with sepsis: a retrospective cohort study

**DOI:** 10.1186/s12879-024-09476-w

**Published:** 2024-06-11

**Authors:** Wen-Ming Shao, Lu-Wei Ye, Lu-ming Zhang, Yu-Long Wang, Hui Liu, Dan He, Jia-Liang Zhu, Jun Lyu, Haiyan Yin

**Affiliations:** 1 Emergency Department, The First Affiliated Hospital of Jinan University, Guangzhou, China; 2 Department of Intensive Care Unit, The First Affiliated Hospital of Jinan University, Guangzhou, China; 3 Guangzhou Women and Children’s Medical Center, Guangzhou Medical University, Guangzhou, Guangdong, China; 4 Department of Anaesthesiology, Hengyang Maternal and Child Health Hospital, Hengyang, China; 5 Department of Clinical Research, The First Affiliated Hospital of Jinan University, Guangzhou, China

**Keywords:** Sepsis, HGB, Long-term, MIMIC-IV database, Propensity score matching

## Abstract

**Background:**

Sepsis is a common and severe disease with a high mortality rate in intensive care unit (ICU). The hemoglobin (HGB) level is a key parameter for oxygen supply in sepsis. Although HGB is associated with the progression of inflammation in sepsis patients, its role as a marker following sepsis treatment remains unclear. Here, we studied the correlation between early temporal changes in HGB levels and long-term mortality rates in septic patients.

**Method:**

In this retrospective study of data on patients with sepsis from the Medical Information Mart for Intensive Care (MIMIC) IV database, the outcome was long-term mortality. Patients were divided based on the cut-off of the HGB percentage for receiver operating characteristic (ROC) curve calculation. Kaplan–Meier (KM) survival curves and Cox proportional hazards regression models were used to analyse the associations between groups and outcomes. Propensity score matching (PSM) was used to verify the results.

**Results:**

In this study, 2042 patients with sepsis and changes in HGB levels at day 4 after admission compared to day 1 were enrolled and divided into two groups: group 1 (n = 1147) for those with reduction of HGB < 7% and group 2 (n = 895) for those with dropping ≥ 7%. The long-term survival chances of sepsis with less than a 7% reduction in the proportion of HGB at day four were significantly higher than those of patients in the group with a reduction of 7% or more. After adjusting for covariates in the Cox model, the hazard ratios (HRs) with 95% confidence intervals (CIs) for long-term all-cause mortality in the group with a reduction of 7% or more were as follows: 180 days [HR = 1.41, 95% CI (1.22 to 1.63), P < 0.001]; 360 days [HR = 1.37, 95% CI (1.21 to 1.56), P < 0.001]; 540 days [HR = 1.35, 95% CI (1.20 to 1.53), P < 0.001]; 720 days [HR = 1.45, 95% CI (1.29 to 1.64), P < 0.001]. Additionally, the long-term survival rates, using Kaplan–Meier analysis, for the group with a reduction of 7% or more were lower compared to the group with less than 7% reduction at 180 days (54.3% vs. 65.3%, P < 0.001), 360 days (42.3% vs. 50.9%, P < 0.001), 540 days (40.2% vs. 48.6%, P < 0.001), and 720 days (35.5% vs. 46.1%, P < 0.001). The same trend was obtained after using PSM.

**Conclusion:**

A ≥ 7% decrease in HGB levels on Day 4 after admission was associated with worse long-term prognosis in sepsis patients admitted to the ICU.

**Supplementary Information:**

The online version contains supplementary material available at 10.1186/s12879-024-09476-w.

## Introduction

Sepsis is a life-threatening organ dysfunction syndrome [1] instigated by the host's exaggerated response to microbial infections and a dysregulated bodily reaction. Immediate treatment is imperative upon its onset. Annually, approximately 49 million new sepsis cases have emerged globally, resulting in 11 million deaths. This mortality rate constitutes nearly 20% of global fatalities, with complications related to sepsis accounting for an alarming 5.3 million deaths [2, 3]. The rising prevalence of sepsis is set to increase, exacerbated by the ageing global population, and the prolific use of invasive devices. It is also among the conditions incurring the highest medical costs [2]. Recognizing its importance, in 2017, the World Health Organization (WHO) explicitly prioritized sepsis as a focal point in global health prevention and care. Identification of early risk factors for sepsis and timely interventions to prevent its progression are urgently needed [4].

Sepsis often presents with a multitude of complications, among which anaemia is one of the most prevalent. The onset and progression of sepsis delineate a complex pathophysiological trajectory. Within this framework, pathogens stimulate the body's inflammatory immune system, modulating the functionality of endothelial cells, coagulation processes, immunity, and hormonal responses.

The pathophysiological mechanisms through which sepsis induces anaemia are multifaceted. These events include an increase in inflammation-inducing factors during sepsis that augment hepcidin, thereby limiting iron utilization [5, 6]; haemodilution associated with fluid resuscitation; iatrogenic blood loss [7]; diminished erythropoietin (EPO) synthesis [8]; and a reduction in red blood cell lifespan [9, 10] and drug suppression-associated anaemia [11]. Additionally, sepsis induces the rupture of red blood cells, which release haemoglobin (HGB). This liberated HGB can generate free radicals, damage endothelial cells and activate the inflammatory response system as a damage-associated molecular pattern [12–14].

In the early stages of sepsis, a decrease in HGB may lead to tissue and organ hypoxia, which may be initially insignificant but, as the disease progresses, can lead to extensive tissue and organ damage. In the advanced stages of sepsis, these injuries may worsen rapidly [15, 16], heightening the mortality risk in critically ill patients [17]. Nonetheless, most contemporary studies have focused primarily on the relationship between sepsis and a single measured HGB level, focusing mainly on HGB thresholds and the timing of red blood cell transfusion. A prior prospective study revealed no statistically significant difference in the 30-day mortality rate between a liberal red blood cell transfusion group (with a HGB threshold < 10.0 g/dL) and a restrictive transfusion group (with a HGB threshold < 7.0 g/dL) [18]. Another extensive randomized controlled study revealed that when comparing the liberal red blood cell transfusion group to the restrictive transfusion group, there were no significant differences in metrics such as the 90-day mortality rate, survival rate upon discharge, or incidence of ischaemic events. Even when the follow-up duration was extended to one year, statistical disparities remained absent between the two cohorts [19]. Furthermore, the benefits of setting transfusion thresholds vary across different populations [20]. Restrictive transfusion is not universally appropriate for all patients. Balancing the risks of anaemia and red blood cell transfusion remains one of the prevailing challenges for physicians. Earlier studies have ascertained that a HGB level ≤ 80 g/L measured within 48 h of ICU admission is one of the predictors of long-term mortality in patients with sepsis, suggesting that early amelioration of HGB levels might be beneficial [21]. However, to date, the potential of changes in HGB levels as an evaluative criterion postsepsis treatment has not been determined. Consequently, this research endeavours to elucidate the correlation between the magnitude of change in the HGB percentage after sepsis treatment and the prognosis of long-term all-cause mortality. The aim was to investigate the association between the change in HGB level on Day 4 versus Day 1 after ICU admission in patients with sepsis and the long-term prognosis.

## Method

### Data source

The data used for this retrospective study were retrieved from the Medical Information Mart for Intensive Care [22] (MIMIC-IV 2.0), which comprises clinical data from a custom hospital-wide electronic health record and an ICU-specific clinical information system for more than 40,000 patients who were admitted to the Beth Israel Deaconess Medical Center (BIDMC) in Boston, Massachusetts, USA, between 2008 and 2019 [23]. The database includes detailed information on patient demographics, laboratory test results, medication use, vital signs and disease diagnosis, among others. The database was approved by the Institutional Review Board of the Massachusetts Institute of Technology and Beth Israel Deaconess Medical Center. To protect patient privacy, all private information in the database depository was removed. Thus, informed consent and the ethical approval statement were waived for this study. The study was consistent with the Declaration of Helsinki compliant principles.

### Data extraction

Patients were excluded if they (1) did not have a diagnosis of sepsis according to the Sepsis 3.0 standard [1], (2) were aged < 18 years, (3) had no first- or fourth-day HGB, (4) stayed in the ICU < 96 h, and (5) lacked HGB data. The MIMIC-IV database was extracted using the Structured Query Language (SQL) [24]. HGB data, recorded on the first and fourth days after admission to the ICU, were extracted from MIMIC-IV 2.0. Differences in HGB levels were calculated using the formula: (HGB day4 − HGB day1)/ HGB day1 × 100%. The variables on Day 1 of ICU admission included age, gender, comorbidities, myocardial infarction (MI), congestive heart failure (CHF), peripheral vascular disease (PVD), cerebrovascular disease (CVD), chronic pulmonary disease (CPD), rheumatic disease (RHD), peptic ulcer disease (PUD), dementia, diabetes, liver disease, paraplegia, lactate, norepinephrine on Day 4, input and output on Day 4, renal replacement therapy on Day 4, blood transfusion on Day 4, invasive mechanical ventilation on Day 4, source of infection, SOFA score, Charlson score, HGB on Day 1, HGB on Day 4, length of stay (LOS) hospital and length of stay in the ICU.

### Primary outcomes

The outcomes of this study were the long-term prognosis of patients with sepsis, including all-cause mortality at 180, 360, 540, and 720 days.

### Statistical analysis

In this study, less than 10% of the data were missing for each variable, which we addressed using the random forest imputation method [25]. We generated a receiver operating characteristic (ROC) curve and determined the optimal cut-off point for long-term mortality by utilizing the Youden index of the ROC curves [26]. Subsequently, patients were stratified into two groups according to this optimal cut-off point.

The normally distributed data are expressed as the mean ± standard deviation, and an independent sample Student’s t test was used. The nonnormally distributed data are expressed as the median (M) and the interquartile range (IQR), and the Mann–Whitney U test was used. Enumeration data are expressed as frequencies and/or percentages, and the chi-square test was used to compare groups.

The Kaplan–Meier (K-M) method was used to draw cumulative incidence curves showing the occurrence of deaths in different groups of sepsis patients at follow-up, and the log-rank test was used to compare the differences in risk between the different groups. It is worth mentioning here that in MIMIC IV 2.0, the maximum time of follow up for each patient is exactly one year after their last hospital discharge [22]. However, survival analysis can handle censored data. A sample that is censored at a certain point in time will not be included in calculations made by survival analysis after that point in time. In other words, a sample that is censored will no longer have an impact on subsequent calculations, but will still provide valuable information for calculations made prior to the censoring. Furthermore, we performed univariate Cox regression analysis and identified the variables with *P* values < 0.1; those variables were then included in our multivariate Cox regression analysis. After adjusting for different covariates, two Cox proportional hazards models were constructed to determine the relationship between changes in the proportion of HGB and patient outcomes. Second, we employed multivariate Cox regression analysis to identify potential confounders (*P* < 0.05). The models include the nonadjusted Model, Model I, and Model II. Model I was adjusted for age and gender, and Model II was adjusted for variables selected by multivariate Cox regression analysis with a significance level of* P* < 0.05. A multivariate Cox regression model was established to assess the independent association between exposure and the primary endpoint.

To ensure that the results were stable and reliable, we further adjusted for covariates using propensity score matching (PSM) and after analysing the original population. The propensity scores were calculated using logistic regression, accounting for clinical characteristics. In the propensity score matching model, variables [27], which included age and gender, comorbidities, ventilation status, transfusion, RRT, vasoactive agent, infection source, and NE, were selected in accordance with a consensus statement from previous literature. A 1:1 ratio was used for matching with a 0.1 calliper [28]. The standardized mean difference (SMD) was calculated before and after matching to assess the difference between the two groups. When the SMD was less than 0.1, a balance was considered reached between the groups [29]. All the statistical analyses in this study were performed using R software (version 4.1.0).

## Results

### Baseline characteristics

**Fig. 1:**
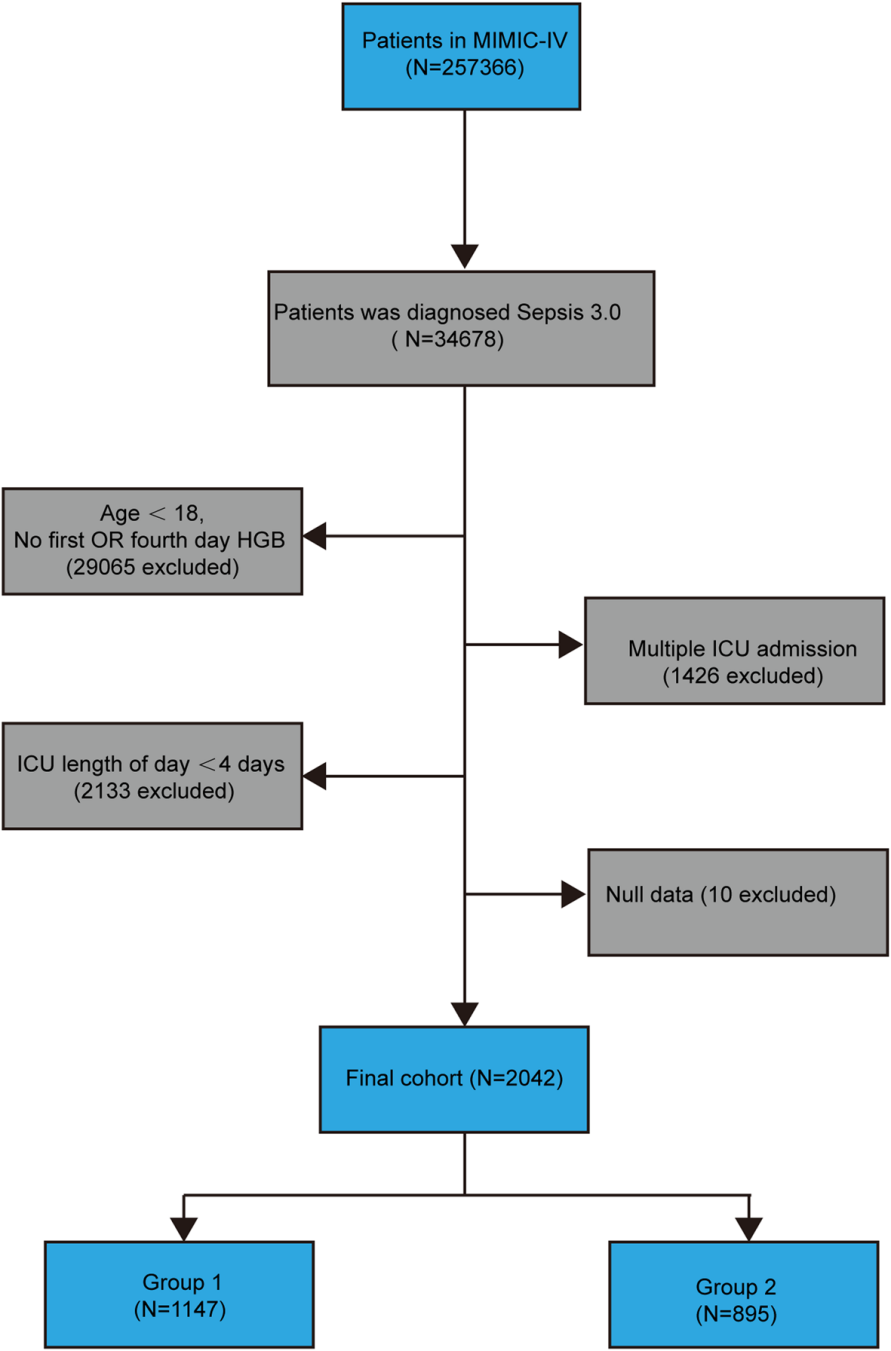
Study flow diagram depicting exclusion criteria and outcomes. Abbreviations: *MIMIC* medical information mart for intensive care, *ICU* intensive care unit, *HGB* haemoglobin, *Group 1*: < 7% Haemoglobin decrease, *Group 2*: > 7% Haemoglobin decrease

The MIMIC-IV database comprises data on 257,366 critically ill patients, 34,678 of whom were diagnosed with sepsis. Our study included 2,042 patients with sepsis based on the inclusion and exclusion criteria (Fig. 1). The optimal cut-off value obtained based on the Yoden index of the ROC curve is -7.3% (almost equivalent to -7%). The study population was categorized into two groups according to the cut-off (< 7%). Patients with sepsis with changes in HGB levels were enrolled on Day 4 after admission. The < 7% Haemoglobin decrease was defined as group 1 and the > 7% Haemoglobin decrease was defined group 2. The group 1 consisted of 1,147 sepsis patients, while the group 2 had 895. A comparative analysis of the baseline characteristics of both groups was conducted both before and after PSM, as shown in Table 1. In the PSM cohort, 895 patients with an exposure < 7% were paired with an equal number of patients in the ≥ 7% category at a 1:1 ratio. Table 1 shows that the covariates in the matching cohort were evenly distributed between the group 1 and group 2, with almost all the covariate SMDs being less than 0.1.

**Table 1:** Baseline characteristics before and after matching

	Before Matching				After Matching				
Characteristics	Group1 (n = 1147)	Group2 (n = 895)	SMD	*p*	Group1 (n = 895)	Group2 (n = 895)	SMD	*p*	Missing data (%)
Age (year)	61.8 ± 14.7	62.6 ± 13.7	0.057	0.219	62.0 ± 14.8	62.6 ± 13.7	0.044	0.372	0.0
Gender, n (%)	480 (41.8)	353 (39.4)	-0.049	0.292	358 (40)	353 (39.4)	-0.011	0.847	0.0
Comorbidities, n (%)									
MI	153 (13.3)	120 (13.4)	0.002	1.000	112 (12.5)	120 (13.4)	0.026	0.622	0.0
CHF	345 (30.1)	319 (35.6)	0.116	0.009	314 (35.1)	319 (35.6)	0.012	< 0.001	0.0
PVD	87 (7.6)	78 (8.7)	0.040	0.396	67 (7.5)	78 (8.7)	0.044	0.386	0.0
CVD	152 (13.3)	133 (14.9)	0.045	0.329	133 (14.9)	133 (14.9)	0.000	1.000	0.0
CPD	299 (26.1)	218 (24.4)	-0.040	0.406	231 (25.8)	218 (24.4)	-0.034	0.513	0.0
RHD	43 (3.7)	49 (5.5)	0.076	0.079	41 (4.6)	49 (5.5)	0.039	0.449	0.0
PUD	101 (8.8)	19 (2.1)	-0.464	<0.001	15 (1.7)	19 (2.1)	0.031	0.603	0.0
Dementia	20 (1.7)	9 (1)	-0.074	0.226	9 (1%)	9 (1)	0.000	1.000	0.0
Liver disease	208 (18.1)	187 (20.9%)	0.068	0.131	158 (17.7%)	168 (18.8)	0.038	0.582	0.0
Diabetes	285 (24.8)	168 (18.8)	-0.156	0.001	58 (6.5)	58 (6.5)	0.029	1.000	0.0
Paraplegia	64 (5.6)	58 (6.5)	0.037	0.448	173 (19.3)	187 (20.9)	0.000	0.443	0.0
Renal disease	212 (18.5)	181 (20.2)	0.043	0.351	177 (19.8)	181 (20.2)	0.019	0.859	0.0
Ventilation_status, n (%)				<0.001				<0.001	9.4
Invasive vent	68 (5.93)	67 (7.49)	0.059		58 (6.48)	67 (7.49)	0.038		
Supplemental oxygen	685 (59.72)	561 (62.68)	0.061		521 (58.21)	561 (62.68)	0.092		
Tracheostomy	115 (10.03)	36 (4.02)	-0.306		87 (9.72)	36 (4.02)	-0.290		
Other	279 (24.32)	231 (25.81)	0.034		229 (25.59)	231 (25.81)	0.005		
Transfusion, n (%)	23 (2.01)	48 (5.36)	0.149	<0.001	21 (2.35)	48 (5.36)	-0.134	<0.001	0.0
RRT, n (%)	9 (0.78)	14 (1.56)	0.063	0.098	9 (1.01)	14 (1.56)	0.045	0.294	1.1
Vasoactive_agent, n (%)	396 (34.52)	318 (35.53)	0.021	0.636	333 (37.21)	318 (35.53)	0.012	0.461	0.0
Infection_source, n (%)				0.041				0.270	0.0
Respiratory	194 (16.91)	117 (13.07)	-0.114		138 (15.42)	117 (13.07)	-0.070		
Blood	114 (9.94)	109 (12.18)	0.068		86 (9.61)	109 (12.18)	0.079		
Stool	25 (2.18)	26 (2.91)	0.043		22 (2.46)	26 (2.91)	0.027		
Urine	71 (6.19)	69 (7.71)	0.057		63 (7.04)	69 (7.71)	0.025		
Other	743 (64.78)	574 (64.13)	-0.013		586 (65.47)	574 (64.13)	-0.028		
NE, n (%)	295 (25.72)	236 (26.37)	0.015	0.740	254 (28.38)	236 (26.37)	-0.046	0.340	0.0
Charlson score	6.0 ± 2.6	6.1 ± 2.5	0.067	0.142	6.0 ± 2.6	6.1 ± 2.5	0.071	0.348	0.0
SOFA score	4 (3, 6)	4 (2, 6)	-0.093	0.045	4 (2, 6)	4 (2, 6)	-0.055	0.159	0.0
Output (ml)	2250 (1320, 3370)	2125 (1145, 3320)	-0.001	0.018	2375 (1374, 3370)	2125 (1145, 3320)	-0.016	0.002	0.5
Input (ml)	2970 (2070, 4317)	2702 (1732, 3956)	-0.116	<0.001	2981 (1943, 4317)	2702 (1732, 3956)	-0.101	0.001	2.2
Lactate (mmol/L)	1.74 ± 0.79	1.56 ± 0.65	-0.266	<0.001	1.72 ± 0.79	1.56 ± 0.65	-0.237	<0.001	9.8
HGB on Day 1 (g/dL)	11.01 ± 1.93	8.34 ± 1.81	-1.474	<0.001	10.94 ± 1.93	8.34 ± 1.81	-1.473	<0.001	0.0
HGB on Day 4 (g/dL)	8.41 ± 1.37	9.15 ± 1.54	0.457	<0.001	8.38 ± 1.34	9.15 ± 1.54	0.499	<0.001	0.0
LOS hospital (day)	40 (25, 72)	42 (22, 57)	-0.162	0.019	39 (24, 72)	42 (22, 57)	-0.63	0.049	0.0
LOS ICU (day)	11 (7, 18)	9 (6, 14)	-0.291	<0.001	11 (7, 17)	9 (6, 14)	-0.276	<0.001	0.0
Mortality									
28 days	191 (16.7)	146 (16.3)	-0.019	0.838	146 (16.3)	146 (16.3)	0.000	>0.999	0.0
60 days	303 (26.4)	229 (25.6)	-0.005	0.672	229 (25.6)	229 (25.6)	0.000	>0.999	0.0
90 days	350 (30.5)	271 (30.3)	-0.009	0.909	271 (30.3)	271 (30.3)	0.000	>0.999	0.0

Data are presented as mean ± standard deviation, median (interquartile range), or number (percent). Group 1: < 7% Haemoglobin decrease, Group 2: > 7% Haemoglobin decrease

Abbreviations: *PSM* propensity score matching, *SMD* standardized mean difference, *MI* myocardial infarction, *CHF* congestive heart failure, *PVD* peripheral vascular disease, *CVD* cerebrovascular disease, *CPD* chronic pulmonary disease, *RHD* rheumatic disease, *PUD* peptic ulcer disease, *RRT* renal replacement therapy, *NE* norepinephrine, *LOS* lengths of stay, *SOFA* sequential organ failure assessment, *ICU* intensive care unit, *HGB* haemoglobin

### Survival analysis

**Fig. 2:**
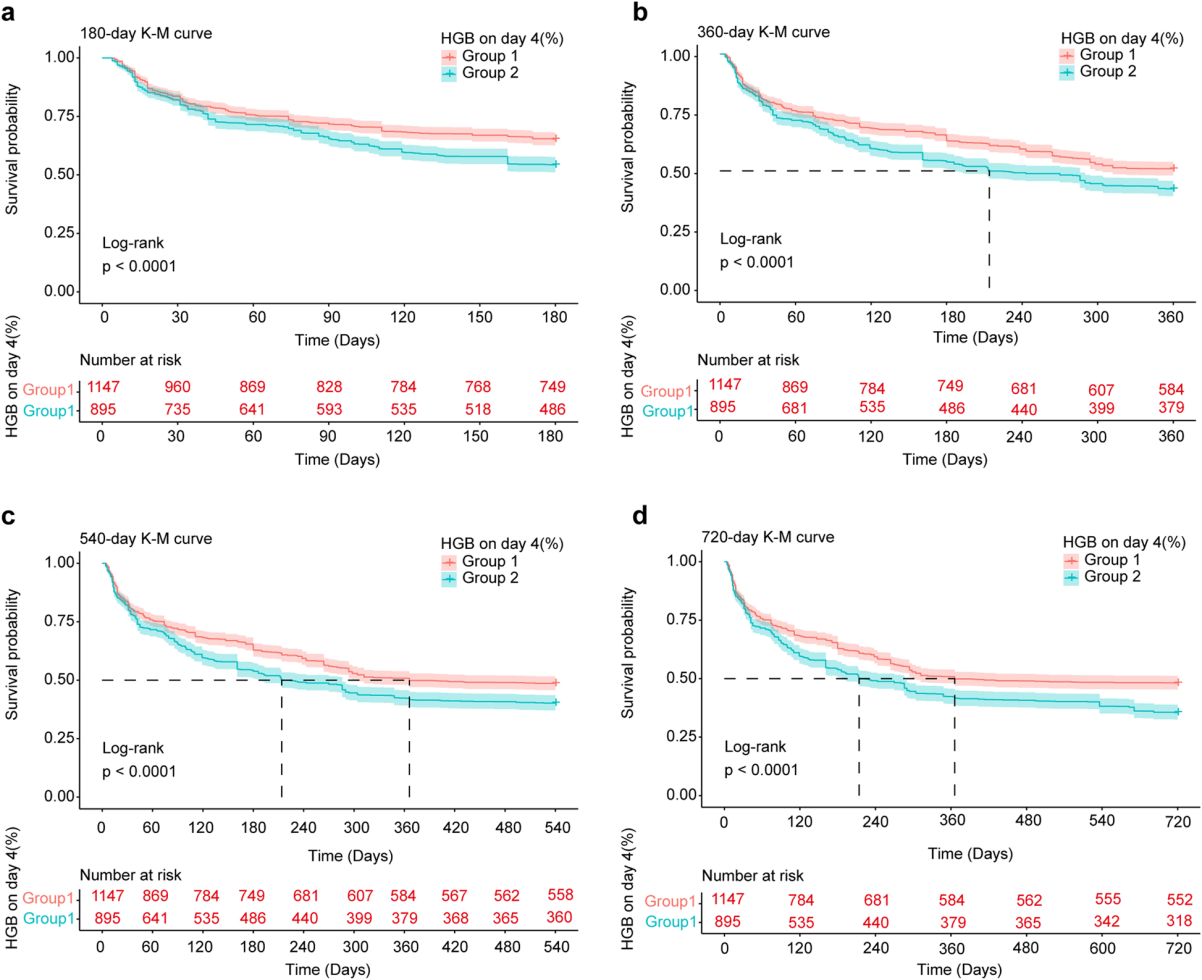
Kaplan‒Meier survival curves of the groups. All-cause mortality before matching was significantly lower in the group 1 than in the group 2 at 180 days, 360 days, 540 days and 720 days (**A**, **B**, **C**, **D**). Abbreviations: *K-M* kaplan–meier, *HGB* haemoglobin, *Group 1*: < 7% Haemoglobin decrease, *Group 2*: > 7% Haemoglobin decrease

**Fig. 3:**
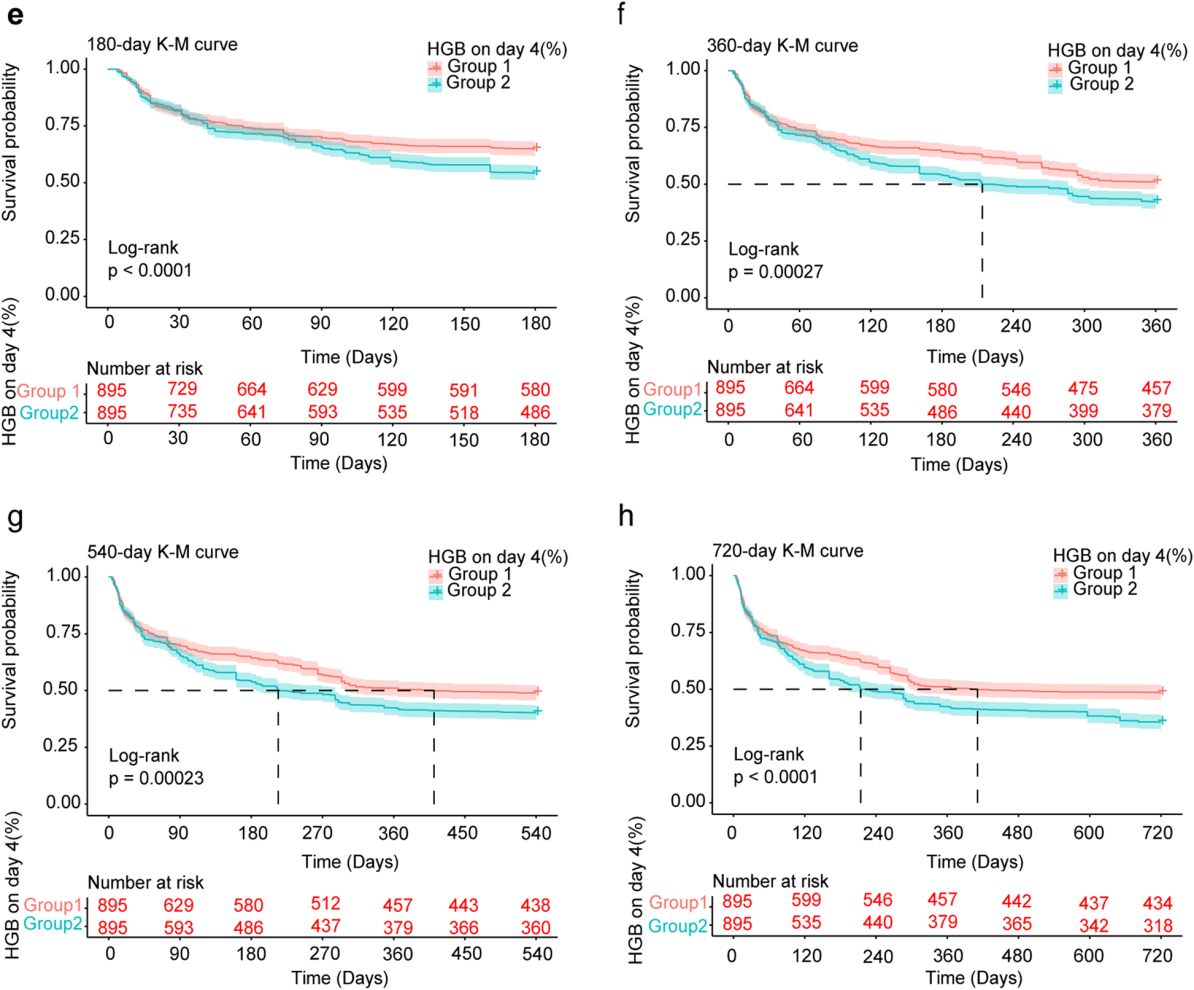
K‒M survival curves of the groups. All-cause mortality postmatching was significantly lower in the group 1 than in the group 2 at 180 days, 360 days, 540 days and 720 days (**E**, **F**, **G**, **H**). Abbreviations: *K-M* kaplan–meier, *HGB* haemoglobin, *Group 1*: < 7% Haemoglobin decrease, *Group 2*: > 7% Haemoglobin decrease

Endpoint events for two groups of patients with sepsis were observed at 180, 360, 540, and 720 days.. In this study, time nodes 180 and 360 days do not exist where the survival status of the patient is unknown. For days 540 and 720, we calculated the proportion of the study sample where this occurred as 21% and 23.947%, respectively. Prior to PSM, Kaplan‒Meier survival estimates revealed that the survival rates for the group with group 1 survival were consistently greater than those for the group with a survival rate group 2. Specifically, the 180 days survival rates were 749 (65.3%) for group 1 and 486 (54.3%) for group 2. The 360 days survival rates were 584 (50.9%) for group 1 and 379 (42.3%) for group 2. The 540 days survival rates were 558 (48.6%) for group 1 and 360 (40.2%) for group 2. Finally, the 720-day survival rates were 552 (46.1%) and 318 (35.5%), and after applying PSM, the outcomes were approximately similar, as shown in Table 2. The Kaplan‒Meier survival curve analysis revealed a marked discrepancy in survival likelihood between the two cohorts. After 180 days, 360 days, 540 days, and 720 days, the likelihood of survival was substantially greater in the group 1 cohort than in the group 2 cohort (Fig. 2). The log-rank test results confirmed the disparity in mortality risk between the two groups, with a significance level of *P* < 0.05. Post-PSM, the outcomes remained consistent with those observed prior to PSM (Fig. 3).

**Table 2:** Survival rates (Kaplan–Meier estimates) for sepsis HGB on Day 4 (%)

Characteristic	180 days (%)	360 days (%)	540 days (%)	720 days (%)
Before PSM				
Overall	1235 (60.4)	963 (47.2)	918 (45.0)	870 (42.6)
Group1	749 (65.3)	584 (50.9)	558 (48.6)	552 (46.1)
Group2	486 (54.3)	379 (42.3)	360 (40.2)	318 (35.5)
After PSM				
Overall	1066 (59.6)	836 (46.7)	798 (44.6)	756 (42.2)
Group1	580 (64.8)	51% (51.1)	438 (48.9)	434 (48.4)
Group2	486 (54.3)	379 (42.3)	360 (40.2)	318 (35.5)

*Group 1:* < 7% Haemoglobin decrease, *Group 2:* > 7% Haemoglobin decrease; *HGB* haemoglobin, *PSM* propensity score matching

### Cox Proportional hazards regression model and propensity score matching

Utilizing both univariate and multivariate Cox regression analyses, the results revealed that confounding factors prior to PSM included age, gender, CVD status, RHD status, ventilation status, transfusion status, RRT, infection source, NE status, Charlson score, SOFA score, lactate level, HGB on Day 1, and length of hospital stay. After PSM, the confounders were age, gender, CVD status, dementia status, RHD status, ventilation status, transfusion status, RRT, infection source, NE, Charlson score, SOFA score, lactate concentration, HGB on Day 1, length of hospital stay, and length of ICU stay (Fig. S1 and Fig. S2). Although gender and NE had P values greater than 0.1, they are conventionally recognized as confounding variables and thus were incorporated into the adjustment factors. According to the multivariate Cox regression model, within 180 days, 360 days, 540 days, and 720 days, the all-cause mortality rate in the group 2 cohort consistently exceeded that in the group 1 cohort. According to the adjusted model, comparisons were made between the group 2 and the group 1 at 180 days, 360 days, 540 days, and 720 days. After nonadjusting for any factors, the results were as follows: [HR = 1.39, 95% CI (1.21 to 1.60),* P* < 0.001]; [HR = 1.28, 95% CI (1.14 to 1.44), *P* < 0.001]; [HR = 1.27, 95% CI (1.13 to 1.43), *P* < 0.001]; and [HR = 1.37, 95% CI (1.22 to 1.54), *P* < 0.001]. According to the adjusted Model I, the results were as follows: [HR = 1.39, 95% CI (1.21 to 1.59), *P* < 0.001]; [HR = 1.27, 95% CI (1.12 to 1.43), *P* < 0.001]; [HR = 1.26, 95% CI (1.12 to 1.42); *P* < 0.001]; and [HR = 1.35, 95% CI (1.21 to 1.52); and *P* < 0.001]. According to the adjusted Model II, the results were as follows: [HR = 1.41, 95% CI (1.22 to 1.63); *P* < 0.001]; [HR = 1.37, 95% CI (1.21, 1.56); *P* < 0.001]; [HR = 1.35, 95% CI (1.20 to 1.53; *P* < 0.001]; and [HR = 1.45, 95% CI (1.29 to 1.64), *P* < 0.001] (Table 3). The results of propensity score matching (PSM) were consistent with the results of prepropensity score matching (Table 3).

**Table 3:** Results of Cox proportional hazard models

HGB on day 4 (%)	Non-Adjust		Model I		Model II	
	HR (95% Cl)	*P* value	HR (95% Cl)	*P* value	HR (95% Cl)	*P* value
**Before PSM**						
180 days mortality						
Group1	Reference		Reference		Reference	
Group2	1.39 (1.21 to 1.60)	<0.001	1.39 (1.21 to 1.59)	<0.001	1.41 (1.22 to 1.63)	<0.001
360 days mortality						
Group1	Reference		Reference		Reference	
Group2	1.28 (1.14 to 1.44)	<0.001	1.27 (1.12 to 1.43)	<0.001	1.37 (1.21 to 1.56)	<0.001
540 days mortality						
Group1	Reference		Reference		Reference	
Group2	1.27 (1.13 to 1.43)	<0.001	1.26 (1.12 to 1.42)	<0.001	1.35 (1.20 to 1.53)	<0.001
720 days mortality						
Group1	Reference		Reference		Reference	
Group2	1.37 (1.22 to 1.54)	<0.001	1.35 (1.21 to 1.52)	<0.001	1.45 (1.29 to 1.64)	<0.001
**After PSM**						
180 days mortality						
Group1	Reference		Reference		Reference	
Group2	1.63 (1.40 to 1.90)	<0.001	1.63 (1.40 to 1.90)	<0.001	1.67 (1.43 to 1.96)	<0.001
360 days mortality						
Group1	Reference		Reference		Reference	
Group2	1.43 (1.26 to 1.63)	<0.001	1.42 (1.25 to 1.62)	<0.001	1.58 (1.38 to 1.81)	<0.001
540 days mortality						
Group1	Reference		Reference		Reference	
Group2	1.41 (1.24 to 1.60)	<0.001	1.40 (1.24 to 1.60)	<0.001	1.54 (1.35 to 1.76)	<0.001
720 days mortality						
Group1	Reference		Reference		Reference	
Group2	1.51 (1.33 to 1.71)	<0.001	1.50 (1.32 to 1.10)	<0.001	1.65 (1.45 to 1.88)	<0.001

*Group 1: *<7% Haemoglobin decrease, *Group 2: *>7% Haemoglobin decrease.

*HGB *haemoglobin, *HR *hazard ratio, *CI *confidence interval,* PSM* propensity score matching

Adjust model for Before PSM:

Non-adjusted model adjust for: None

Adjust I model adjust for: Age, Gender

Adjust II model adjust for: Age, Gender, CVD, RHD, Ventilation status, Transfusion, RRT, Infection source, NE, Charlson score, SOFA score, Lactate, HGB on Day 1, LOS hospital

Adjust model for after PSM

Non-adjusted model adjust for: None

Adjust I model adjust for: Age, Gender

Adjust II model adjust for: Age, Gender, CVD, Dementia, RHD, Ventilation_status, Transfusion, RRT, Infection_source, NE, Charlson score, SOFA. score, Lactate, HGB on Day 1, LOS hospital, LOS ICU

## Discussion

In this retrospective cohort study, we analysed 2,042 patients with sepsis and observed that a decrease in HGB level of group 2 between the first and fourth days of ICU stay was correlated with long-term all-cause mortality in patients with sepsis. These findings suggest that the proportional change in HGB levels can serve as a one indicator of long-term prognosis in sepsis. Thus, for patients with sepsis admitted to the intensive care unit (ICU), a decrease in HGB levels on Day 4 after admission of 7% or more serves as an indicator of long-term prognosis. In addition, we briefly investigated the association between changes in HGB and short-term prognosis such as 28-day mortality (Fig. S3). Different from the results of long-term prognosis, changes in HGB were not associated with short-term prognosis. The short-term unfavourable prognostic outcomes of this study align with those reported in previous research [30], which concluded that there is no significant difference in patient survival rates within the short-term period (28/30 days) between liberal and restrictive red blood cell transfusion strategies in the context of sepsis or septic shock.

HGB serving as a crucial vector for oxygen transport, has its levels directly impacting the supply of oxygen to tissues. Reduced levels of HGB may lead to a decrease in oxygen carriage capacity, which in turn can precipitate tissue hypoxia, affecting cellular biofunctionality, manifesting specifically as microcirculatory and metabolic disturbances [16, 31].Previous study [32] have indicated that in the early stages of sepsis, organ and tissue dysfunction is observed and may worsen the long-term prognosis of sepsis patients, which is closely associated with tissue hypoxia caused by low HGB levels. Consistent with these findings, our study results demonstrate a correlation between an early decrease in ICU patient hemoglobin levels by 7% or more and poor long-term outcomes in sepsis. Patients with sepsis frequently exhibit anaemia. This is predominantly attributed to sepsis stimulating a systemic inflammatory immune response, leading to premature erythrocyte destruction and thereby exposing free HGB. This free HGB precipitates inflammatory reactions, subsequently impairing bodily tissues and organs [33]. Inflammatory mediators prevent erythropoiesis and amplify hepcidin, thereby undermining iron utility, and oxidative stress precipitates premature erythrocyte apoptosis [13, 14]. These multifaceted mechanisms collectively result in diminished HGB concentrations in the bloodstream, leading to compromised oxygen-carrying capacity. This invariably results in systemic tissue and organ hypoxia and dysfunction [34], heralding an adverse clinical prognosis.

The majority of existing research has primarily examined the correlation between a single HGB level measured postadmission and the prognosis of sepsis. However, the literature elucidating the impact of the proportional change in HGB levels on short-term or long-term outcomes is conspicuously scant. It is universally acknowledged that the dynamic monitoring of aberrant clinical indicators to guide diagnostic and therapeutic measures holds profound clinical significance. Thus, this study sought to discern the relationship between the variance in HGB levels on the fourth day relative to the first day post-ICU admission and the long-term prognosis in sepsis patients.

Previous research involving 235 patients with sepsis indicated that HGB levels ≤ 80 g/L, measured within the first 48 h of ICU admission, were a risk factor for all-cause mortality during a one-year follow-up period [21]. These findings underscore the clinical imperative for early targeted intervention regarding HGB levels to mitigate the long-term adverse outcomes of sepsis. A separate investigation [35]encompassing 815 patients with sepsis suggested that an admission HGB level less than 10 g/L is associated with an elevated risk of in-hospital mortality, with anaemia in patients with sepsis doubling postadmission. For patients explicitly diagnosed with sepsis, early preventative measures against concurrent anaemia might improve the prognosis.

Our current findings suggest that a ≥ 7% decrease in the magnitude of HGB change on the fourth day after ICU admission in patients with sepsis is an associated risk factor for the prognosis of long-term all-cause mortality in patients with sepsis. After we adjusted for all potential confounders via Cox regression analysis, the mortality rate in the group 2 was markedly greater than that in the group 1 (180 days, HR = 1.41, 95% CI = 1.22–1.63, *P* < 0.001; 360 days, HR = 1.37, 95% CI = 1.21–1.56, *P* < 0.001; 540 days, HR = 1.35, 95% CI = 1.20–1.53, *P* < 0.001; and 720 days, HR = 1.45, 95% CI = 1.29–1.64, *P* < 0.001). Furthermore, Kaplan‒Meier curves indicated a pronounced disparity in survival rates as the follow-up period increased. Notably, the all-cause mortality rate for patients with sepsis aged between 180 and 360 days was greater than that for patients aged 360 to 720 days. These findings underscore the importance of vigilant monitoring of physiological parameters, especially HGB fluctuations, in patients with sepsis during their first year postdischarge. Research has also indicated that as the change in HGB level group 2, the greater the decrease is, the poorer the clinical outcome. Conversely, a decrease in HGB of group 1 acts as a protective factor against all-cause mortality in patients with sepsis. Hence, when the decrease in the HGB concentration surpasses 7%, clinicians might need to contemplate interventional measures or intensify baseline treatments. We posit that a proportional decrease in HGB is a pertinent risk factor for all-cause mortality in patients with sepsis. We believe that sepsis may be caused by a systemic excessive inflammatory response and microcirculatory disorders combined with insufficient oxygen supply, increased oxygen consumption, and the presence of oxygen debt [36]; additionally, coupled with a decrease in HGB, "oxygen debt" is further aggravated, leading to extensive tissue and organ damage and further damage to the body, aggravating the progression of sepsis [37].

The HGB concentration is strongly correlated with damage to myocardial and cerebral cells. In another study [38] involving 2,265 patients with septic shock, HGB levels less than 90 g/L were associated with sepsis-related mortality. This evidence highlights the adverse prognostic relevance of diminished HGB levels in sepsis outcomes. Therefore, intensive care unit (ICU) clinicians must diligently monitor HGB levels in sepsis patients, observe dynamic fluctuations in HGB levels, and, when necessary, intervene promptly to prevent potential disease exacerbation.

## Strengths and Limitations

The distinctive strengths of our research include the use of an expansive cohort of 2,042 patients with sepsis from a public database. Furthermore, we employed multivariate Cox Proportional hazards regression model, propensity score matching, doubly robust procedures, and nuanced subgroup analyses to evaluate the integrity and reliability of our findings. This research has several limitations. First, the study focused on patients with sepsis; its applicability to other demographic conditions requires further clinical validation and investigation. Second, this study focused on the relationship between HGB changes and long-term prognosis of sepsis patients. Based on the fact that this study was a single-center retrospective study, more studies are needed to investigate the association between the level of HGB changes and long-term prognosis of sepsis patients. Meanwhile, the relationship between HGB change levels and short-term prognosis of sepsis patients, such as 28-day mortality and ICU mortality, also needs to be explored in more studies. Third, although our results exhibit consistency and account for confounding factors, there is inescapable potential for selection and confounding biases. The database also includes omissions or exclusions exceeding 20% for some indicators; hence, limited covariates were included, and some key indicators affecting outcomes were not recorded or had large missing values, such as procalcitonin and C-reactive protein. In subsequent steps, we aimed for comprehensive clinical data collection related to outcomes. Fourth, given the observational nature of this research, the observed results merely illustrate phenomena and fail to establish a causal relationship. Variations in HGB levels might either activate or inhibit specific mechanisms, necessitating further research to ascertain and validate potential influential mechanisms. Fifth, this study did not consider changes in lactate, which are more responsive to changes in tissue hypoxia in patients, and it is hoped that they will be considered in parallel in future studies. However, our utilization of the extensive MIMIC-IV database offers valuable insights for revealing potential underlying mechanisms in sepsis and paves the way for prospective research.

## Conclusion

In conclusion, a ≥ 7% decrease in HGB levels on Day 4 after admission was associated with worse long-term prognosis in sepsis patients admitted to the ICU.

### Supplementary Information

Supplementary Material 1: Fig. S1 Abbreviations: *PSM* propensity score matching, *SMD* standardized mean difference, *MI* myocardial infarction, *CHF* congestive heart failure, *PVD* peripheral vascular disease, *CVD* cerebrovascular disease, *CPD* chronic pulmonary disease, *RHD* rheumatic disease, *PUD* peptic ulcer disease, *RRT* renal replacement therapy, *NE* norepinephrine, *LOS* lengths of stay, *SOFA* sequential organ failure assessment, *ICU* intensive care unit, *HGB* haemoglobin.

Supplementary Material 2: Fig.S2 Abbreviations: *PSM* propensity score matching, *SMD* standardized mean difference, *MI* myocardial infarction, *CHF* congestive heart failure, *PVD* peripheral vascular disease, *CVD* cerebrovascular disease, *CPD* chronic pulmonary disease, *RHD* rheumatic disease, *PUD* peptic ulcer disease, *RRT* renal replacement therapy, *NE* norepinephrine, *LOS* lengths of stay, *SOFA* sequential organ failure assessment, *ICU* intensive care unit, *HGB* haemoglobin.

Supplementary Material 3: Fig.S3 Kaplan‒Meier survival curves of the groups. All-cause mortality before matching was not significantly for group 1 and for group 2 at 28 days. Abbreviations: *K-M* kaplan–meier, *HGB* haemoglobin, *Group 1*: < 7% Haemoglobin decrease, *Group 2*: > 7% Haemoglobin decrease.

## Data Availability

Publicly available datasets were analysed in this study. These data can be found here. The data were available on the MIMIC-IV website at https://mimic.mit.edu/.

## Abbreviations

MIMIC-IV: Medical information mart for intensive care-IV
HGB: Haemoglobin
SMD: Standardized mean difference
PSM: Propensity score matching
MI: Myocardial infarction
CHF: Congestive heart failure
PVD: Peripheral vascular disease
CVD: Peripheral vascular disease
CPD: Chronic pulmonary disease
RHD: Rheumatic disease
PUD: Peptic ulcer disease
RRT: Renal replacement therapy
NE: Norepinephrine
LOS: Length of stay
SOFA: Sequential organ failure assessment
ICU: Intensive care unit
HR: Hazard ratio
