# Supplementary figures and images for: Relationship between the magnitude of haemoglobin changes and long-term mortality in patients with sepsis: a retrospective cohort study
Source: BMC Infect Dis. 2024 Jun 11;24:577. doi: 10.1186/s12879-024-09476-w (PMC11167884; doi:10.1186/s12879-024-09476-w)

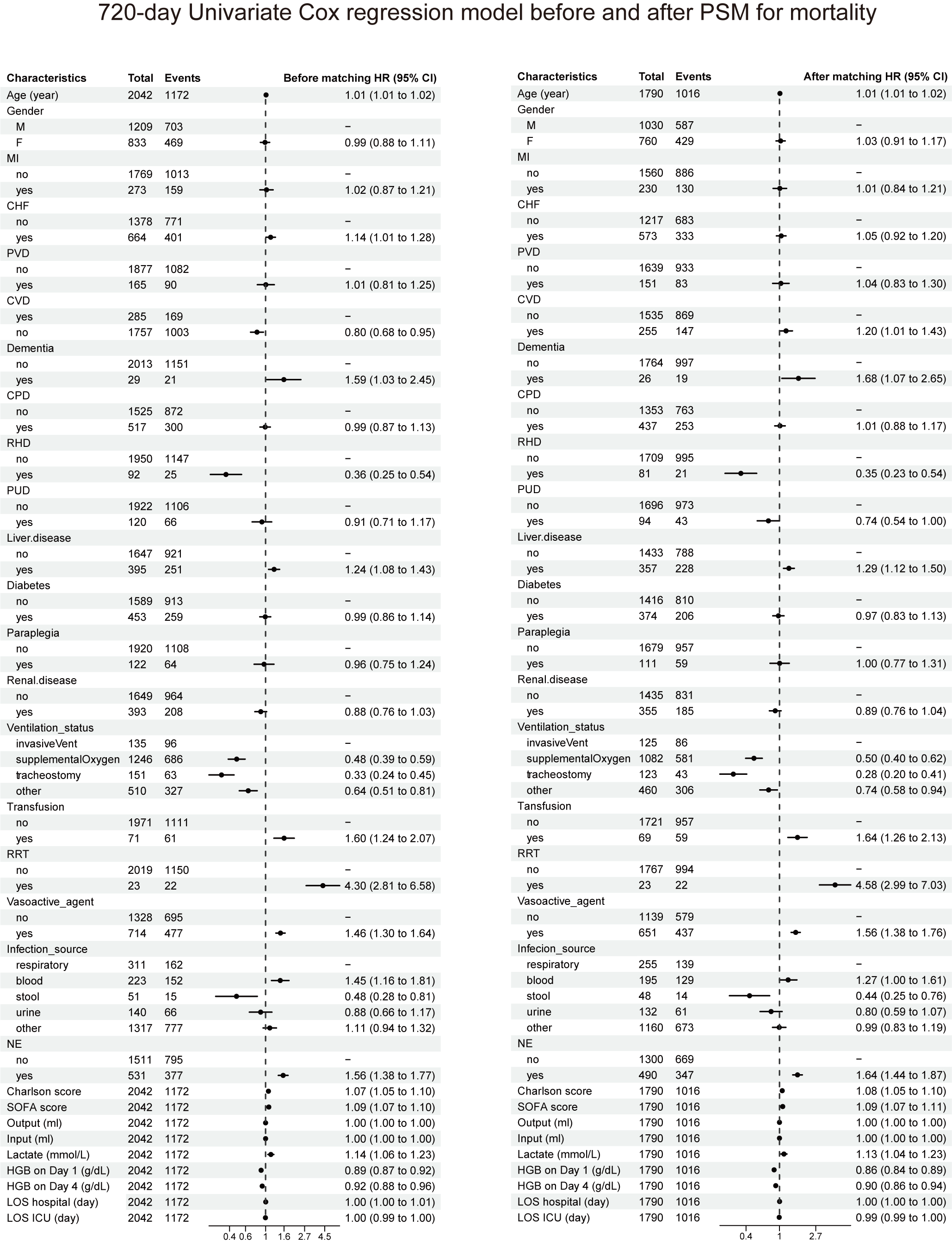

Supplement: Supplementary file 1 — Supplementary Material 1: Fig. S1 Abbreviations: PSM propensity score matching, SMD standardized mean difference, MI myocardial infarction, CHF congestive heart failure, PVD peripheral vascular disease, CVD cerebrovascular disease, CPD chronic pulmonary disease, RHD rheumatic disease, PUD peptic ulcer disease, RRT renal replacement therapy, NE norepinephrine, LOS lengths of stay, SOFA sequential organ failure assessment, ICU intensive care unit, HGB haemoglobin. [file 12879_2024_9476_MOESM1_ESM.tif]

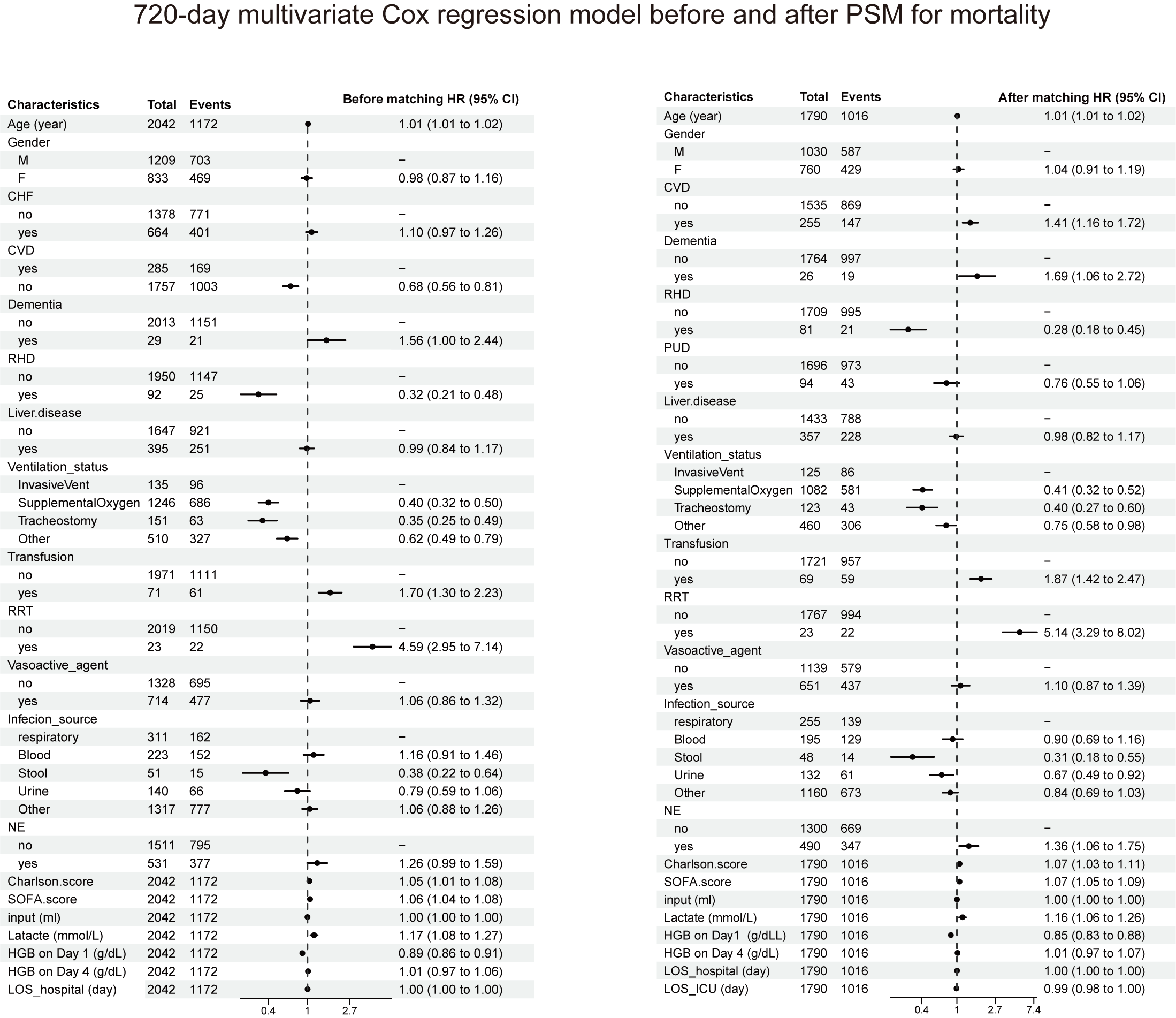

Supplement: Supplementary file 2 — Supplementary Material 2: Fig.S2 Abbreviations: PSM propensity score matching, SMD standardized mean difference, MI myocardial infarction, CHF congestive heart failure, PVD peripheral vascular disease, CVD cerebrovascular disease, CPD chronic pulmonary disease, RHD rheumatic disease, PUD peptic ulcer disease, RRT renal replacement therapy, NE norepinephrine, LOS lengths of stay, SOFA sequential organ failure assessment, ICU intensive care unit, HGB haemoglobin. [file 12879_2024_9476_MOESM2_ESM.tif]

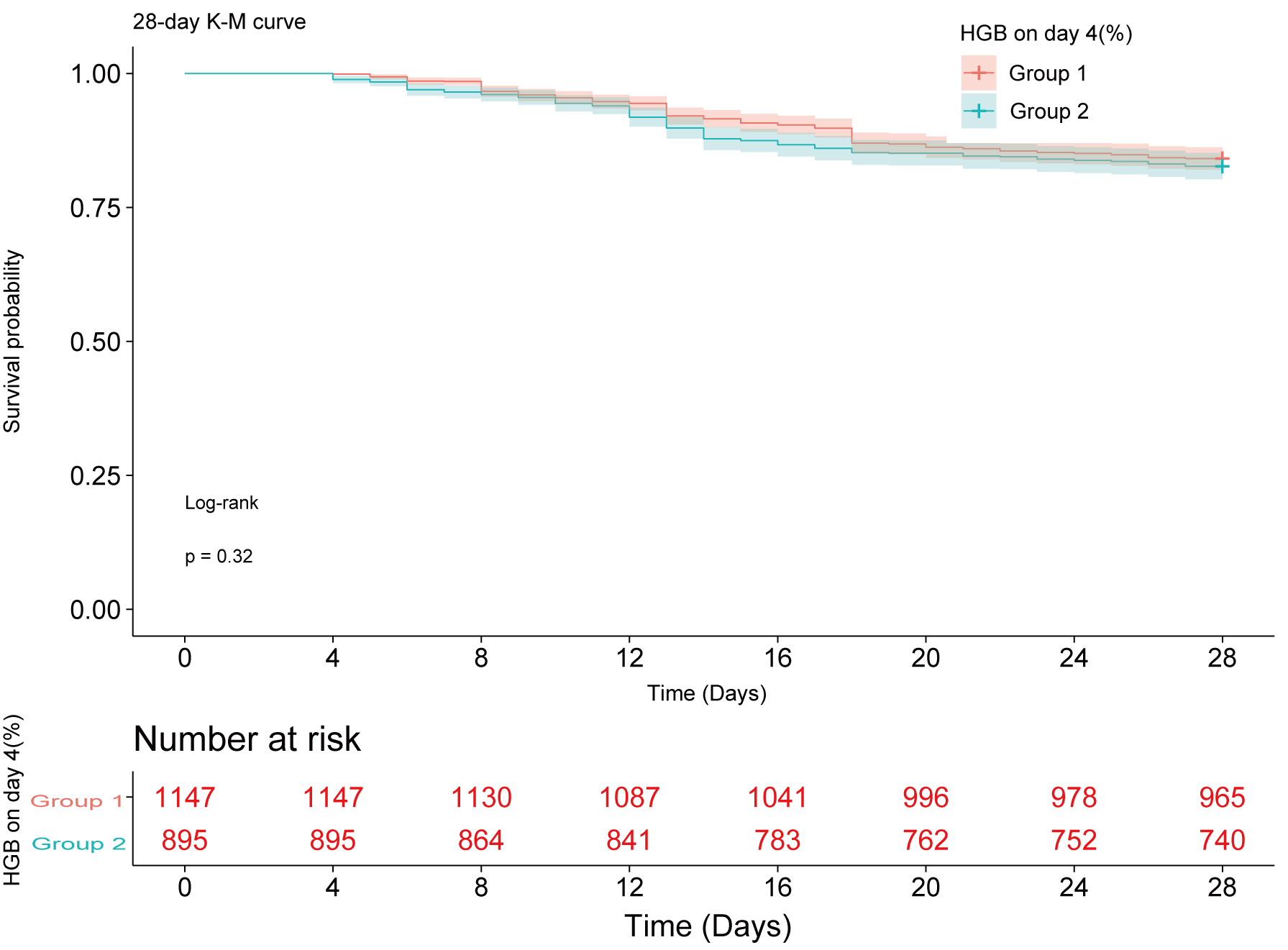

Supplement: Supplementary file 3 — Supplementary Material 3: Fig.S3 Kaplan‒Meier survival curves of the groups. All-cause mortality before matching was not significantly for group 1 and for group 2 at 28 days. Abbreviations: K-M kaplan–meier, HGB haemoglobin, Group 1: < 7% Haemoglobin decrease, Group 2: > 7% Haemoglobin decrease. [file 12879_2024_9476_MOESM3_ESM.tif]
